# Environmental and occupational respiratory diseases - 1054: Sensitization to storage mites, wheat and yeast allergens in Cuban bakers

**DOI:** 10.1186/1939-4551-6-S1-P52

**Published:** 2013-04-23

**Authors:** Mirta Alvarez Castello, Yacquelin Leyva Marquez, Raúl Lázaro Castro Almarales, Alexis Labrada Rosado, Victor R Meli, Humberto Barata

**Affiliations:** 1 Allergology Department, University Hospital General Calixto García, Havana, Cuba, WAO Member, Cuban Society of Allergy , Asthma and Clinical Immunology, Member, Cuban Society of Immunology Member, Havana, Cuba; 2 Allergology Department, Calixto García University Hospital, Havana, Cuba; 3 National Center of Bioproducts, Member of WAO, Cuban Society or Allergy, Cuban Society of Immunology and Cuban Society of Family Medicine , Mayabeque, Cuba; 4 Allergen, Biocen, Cuba; 5 Diater Laboratories, Buenos Aires, Argentina

## Background

For many years, bakers have been considered a professional group at risk of developing allergic diseases by factors related to the work environment. In our country, in this employment sector, the frequency of allergic sensitization to dust mites, wheat flour, soy and yeast is not known. This study aimed at assessing the frequency of work-related symptoms and sensitivity to mites and other occupational allergens among bakery workers through skin prick test (SPT).

## Methods

A no-matched case-controlled study was performed. The study group included 17 bakery workers in Havana, Cuba, median age 33 (range 18- 55 years old), and the control group comprised 14 non-exposed subjects, median age: 28 (range 22-47 years old). Both groups had male predominance. A medical resume and SPT were performed to all subjects.

## Results

Of 17 bakery workers, eight had one or more work-related symptoms and the respiratory symptoms were the most reported. The higher sensitization frequency in bakers corresponded to yeast (88.2 %), followed by storage and house dust mites, *A. siro* and *Dermatophagoides siboney* (a Caribbean species similar to *D. farinae*) and wheat with 82.3% each one. The least sensitization value was found to *D. pteronyssinus* (only 47 %), in contrast to the response pattern found in the control group where this mite was the predominant sensitizing allergen. The largest wheal diameters were found to *A. siro* and *T. putrescientae* in the bakers group and to *D. farinae* and B. *tropicalis* in the control group.

## Conclusions

There is a high sensitivity to storage mites and wheat flour and yeast allergens among bakery workers, which can be an important risk factor to consider for their occupational safety.

